# Enhancing EEG based cognitive state classification using graph Fourier transform

**DOI:** 10.3934/Neuroscience.2026011

**Published:** 2026-05-15

**Authors:** Shweta Sharma, Ayushi Kotwal, Rajneet Kaur Bijral, Vinod Sharma, Jatinder Manhas

**Affiliations:** Department of Computer Science & IT, University of Jammu, Jammu & Kashmir, India

**Keywords:** EEG, graph Fourier transform (GFT), graph signal processing (GSP), machine learning, SHAP

## Abstract

Electroencephalography (EEG) based cognitive state classification has been widely explored with deep-learning models demonstrating remarkable performance. Deep-learning models need high computational resources and large datasets, creating a need for an alternative methodology. Graph signal processing (GSP) techniques provide an effective lightweight alternative by capturing spatial dependencies across the channels. This study investigates the effectiveness of GSP-based graph Fourier transform (GFT) features on a publicly available EEG mental arithmetic task (EEGMAT) dataset. A strict 5-fold cross-validation has been used as an evaluation technique. GFT is applied to extract spatial-spectral features by modeling EEG-channels as graph nodes. The extracted features have been evaluated using multiple classifiers including Random Forest (RF), Extreme Gradient Boosting (XGB), Decision Tree (DT), and Logistic Regression (LR). Statistical analysis confirms the significance of GFT features compared to raw signals. RF gave the highest accuracy of approximately 99 percent. Model interpretability based on Shapley additive explanations (SHAP) revealed that the frontal and central regions contributed to the classification aligning with the findings of cognitive neuroscientists.

## 1. Introduction

Electroencephalography (EEG) is a widely adopted, noninvasive technique that measures electrical activity generated by neuronal oscillations in the brain [1]. Due to its portability, affordable cost, and high temporal resolution, EEG has become a preferred modality for recording brain signals in both research and clinical contexts [2]. Although it suffers from relatively low spatial resolution and produces signals that are inherently nonlinear and non-stationary, EEG remains a powerful tool of monitoring of neural and cognitive processes [3].

EEG has found broad applicability across various domains, generally categorized into three major areas: (i) diagnosis and monitoring of neuropsychological disorders, (ii) cognitive and emotional state assessment, and (iii) brain-computer interface (BCI) development [4]–[6]. A critical step in EEG signal analysis is feature extraction, which plays a pivotal role in transforming raw, noisy signals into meaningful representations for further analysis [7]. Traditional feature extraction techniques—including frequency-domain, time-domain, wavelet based, and entropy-based methods—have provided valuable insights into brain function, cognitive load, emotional states, and neurological disorders [8].

The human brain has two hemispheres: right and left. Further, the brain has four major regions: frontal lobe (behind forehead), occipital lobe (back of the brain), parietal lobe (top middle of the brain), and temporal lobe (near the ears) [9]. Each lobe corresponds to some functional areas of the brain, such as memory, cognition, or perception. To record the brain signals of a subject, the EEG device needs to be attached to the scalp of the subject using electrodes. There exists an international 10–20 system of placement of electrodes where the electrodes are placed such that the distance between the adjacent electrodes is either 10 percent or 20 percent of the total distance between the front to back or left to right portion of the skull [10]. The placement is based on different brain regions. Table 1 explains each brain region with its key functions and electrode placement notations. The electrodes can be placed according the necessity of the experiment.

**Table 1.** Different brain regions and their key function.

Brain Region	EEG electrodes associated	Key functions
Frontal (F)	Fp1, Fp2, F3, F4, F7, F8, Fz	Attention, motor control
Parietal (P)	P3, P4, Pz, P7, P8	Senses, reasoning
Temporal (T)	T3, T7, T4, T8, T5, T6	Hearing, memory, language
Occipital (O)	O1, O2, Oz	Visual Processing
Central region (C)	C3, C4, Cz	Movement, touch
Midline (z)	Fz, Cz, Pz, Oz	Bilateral activity reference point

As raw signals are received from an EEG machine, different feature extraction methods are applied on them before going for further analysis. The brain signals are analyzed by implementing various time- and frequency-based methods, which provide valuable insights to study cognitive states, emotional states, and neurological disorders of the human brain [11]. When it comes to multichannel EEG data, conventional approaches fail to read the spatial relationships and topological structures of the data. To overcome this, spatial-based and graph-based feature extraction techniques are being used to capture spatial, topological, and spectral characteristics of brain activity [12],[13]. Common spatial pattern and spatial filtering are some spatial-based techniques that are able to extract features based on inter-channel dependencies and scalp locations. For graph-based techniques, graph signal processing and graphical neural networks are the methods applied to get functional or statistical connections between the electrodes.

The graph signal processing (GSP) technique provides some powerful tools to analyze the nonlinear and nonstationary EEG signals. They project the EEG channels as graph nodes, making a structural connection between the channels through the edges of the graph. The edges can be defined based on physical proximity, functional connectivity, or statistical relationships of the nodes—electrodes, in the case of EEG analysis. The underlying graph structure with the signal values gives formidable information about the data collected. GFT is one of the fundamental techniques of GSP, decomposing the EEG signals into graph frequency components. It worked on the eigenvectors of the graph Laplacian. GFT provides insight into signal variations over the brain topology and how they are related to the graph structure.

The main contributions of this study are:

i) A systematic evaluation of spatial features extracted from multichannel EEG data for cognitive state classification.

ii) A comprehensive comparison of four classical machine learning algorithms with GFT-based features versus raw signals.

iii) Statistical testing to confirm the superiority of GFT-based features.

iv) Shapley additive explanation (SHAP)-based interpretability to consolidate the relevance of spatial brain regions in cognitive state classification.

## 2. Related study

An extensive exploration of cognitive state classification based on EEG signals has been done by researchers recently, employing various signal processing and artificial intelligence-based methods. These works can be broadly divided as: (i) conventional statistical and frequency-domain methods, (ii) deep learning and ensemble architectures, and (iii) graph-based and connectivity-driven approaches.

Statistical analysis, when combined with conventional methods such as wavelet-based feature extraction, demonstrates promise for tasks like cognitive overload detection. For instance, Zarjam et al. achieved 98% accuracy using wavelet coefficients with a multilayer perceptron (MLP). The model outperformed self-reported measures of mental fatigue, though the absence of standardized experimental protocols (e.g., n-back or SIMKAP tasks) limits reproducibility and generalization [14]. Similarly, Elkerdawy et al. investigated multiple cognitive dimensions—including attention, working memory, and planning—and found random forest (RF) to outperform deep networks, highlighting that classical ensemble methods can still rival deep models in small-sample EEG scenarios [15]. Apicella et al. further demonstrated the utility of support vector machines (SVMs) in classifying cognitive and emotional engagement during e-learning, proving the usability of machine learning (ML) algorithms [16].

Moving towards deep learning and hybrid methods, a Filter-Bank Common Spatial Pattern (FBCSP) pipeline with long short-term memory (LSTM) classifiers was proposed by Chakladar et al., achieving 87% accuracy in mental arithmetic and motor imagery tasks. In a follow-up, the performance of the simultaneous task EEG workload dataset was improved to 92%, implying optimization-driven feature selection, along with an Long Short Term Memory (LSTM)-bidirectional LSTM (BiLSTM) framework [17],[18]. These studies underscore the potential of temporal modeling but also reveal sensitivity to handcrafted preprocessing and feature selection heuristics. Transfer learning approaches, such as Bhuiyan et al.'s application of VGG-16 and ResNet-50 on the Distance Learning dataset, yielded accuracies around 72%, suggesting that pretrained computer vision models may struggle to capture EEG-specific dynamics without domain adaptation [19].

Recent studies have explored feature extraction approaches integrating frequency analysis and deep models. Variational mode decomposition (VMD) has been used for frequency domain analysis. When combined with ML algorithms, VMD yielded good results in emotion detection [20]. Efficient deep-learning-based feature extraction architecture has been applied to EEG-based classification of major depressive disorder. NeuroFeat is one such architecture advancing the state of EEG-based feature learning [21]. Hallal et al. carried out an intensive literature review on deep-learning-based feature extraction for neurocognitive applications using EEG. The study contextualized the trade-off between model complexity and performance [22].

In contrast, graph-based approaches have gained momentum for their ability to model inter-channel dependencies. Sharma and Meena demonstrated that graph Fourier transform (GFT) features did remarkably better than the traditional wavelet features in Alzheimer's classification, achieving 98.9% with decision trees [23]. Jiao et al. successfully leveraged both spectral and temporal dependencies by integrating GFT with BiLSTM to classify epilepsy EEG [24]. Similarly, Ganagapuram et al. applied graph convolutional networks (GCNs) on mental arithmetic tasks, using Bayesian functional connectivity, while Mathur et al. explored joint Laplacian energy features for SVM and decision tree classifiers, reporting robust performance on multichannel setups [25],[26]. Collectively, these studies affirm that graph signal processing (GSP) techniques, particularly GFT, provide a principled framework for incorporating spatial dependencies that conventional methods neglect.

A research gap can be seen with GFT being integrated with deep architectures (e.g., BiLSTM, GCN), but there is limited investigation into its systematic impact on classical supervised learning algorithms, which remain widely used due to interpretability and efficiency. In this study, the GFT features were extracted and classified using different machine learning algorithms. Finally, the results were compared and assessed. A methodological novelty and practical insights into the role of graph spectral representations in EEG cognitive state classification is being offered by this approach.

## 3. Material and methods

EEG signals have found their usability in various domains such as neuropsychological disorders, cognitive assessments, and brain-computer interface. The cognitive state assessment has found vital roles in fields such as emotional assessment and e-learning. The dataset that has been used in this study is the publicly available EEGMAT dataset. This dataset is available on the MIT Physionet [27]. It has the EEG recordings of 36 subjects performing arithmetic tasks of serial subtraction. The data has been collected using 19 EEG channels at the sampling rate of 500 Hz. After the bandpass filtering, the data from 0.4 Hz to 40 Hz has been retained. The subjects are labelled as good (G) or bad (B) depending upon their performance in the arithmetic task. A total of 10 subjects belonged to count quality B, labelled as 0, and 26 belonged to count quality G, labelled as 1, leading to a class imbalance. The count quality was taken as the target label for classification.

In this study, the graph Fourier transform (GFT) is directly applied to the time points of the raw signals recorded with 19-channels. Each subject's recording is preprocessed with a band pass filter of 0.4 Hz and 40 Hz, followed by z-score normalization. The GFT projection yields an N-dimensional graph spectral feature vector per time-point (N is the number of channels, 19 in our case). This vector is then used as an input to the classifiers. No hand-crafted frequency band decomposition or segmentation was applied prior to implementing GFT.

### 3.1. Ethical approval of research

The data was collected anonymously by original authors with the consent of the participants. As no additional human data was collected, no ethical approval was required.

### 3.2. Graph Fourier transform

The EEG channels are irregularly placed on the scalp and the brain activity is not spatially regular, resulting in EEG signals being non-Euclidean. The application of Fourier methods works well on these irregular structures.

In the conventional methods to study EEG signals, the spatial relationships between the brain regions were overlooked. The signals were considered independent and different from the channels they were acquired from. The graph Fourier transform method provides a framework to analyze the brain activity pattern across the network where EEG channels are modeled as the nodes of the graph and their functional connectivity defines the edges. The edges are defined by the connectivity between the channels, which are based on different parameters such as the distance between the electrodes, statistical dependencies, and functional measures.

The graph structure is established based on any of the given parameters, and the graph Laplacian matrix is computed for the obtained graph. EEG signals are projected onto the graph Fourier basis, which is calculated from the eigenvectors of the Laplacian matrix. The projection yields the graph Fourier coefficients, which describes the distribution of signal variations across the graph's spatial nodes.

Suppose there are *n* EEG electrodes used in the experimental setup. Then, the signal at the time point *t* is $x(t)\in {\mathbb{R}}^{n}$, which represents the voltage recorded at the time point *t* by each electrode. For a graph *G* = (*V*, *E*),

V = set of nodes where each EEG electrode is a node,

E = set of edges representing relation between EEG electrodes.

For the graph *G*, the adjacency matrix $A,A_{i,j}=corr\left(x_{i}\left(t\right),x_{j}\left(t\right)\right)$and the degree matrix $D,D_{ii}=\overset{N}{\underset{j=1}{\sum }}A_{ij}$ are calculated.

The graph Laplacian *L* = *D* − *A* is calculated to get a picture of how each electrode is connected to the others. The eigenvalues *λ_i_* and eigenvectors *µ_i_* of the Laplacian L gives an overview of graph-based spatial frequencies and spatial frequency modes, respectively. Eigenvectors explain the spatial changes in the EEG signals across the scalp. The variation of eigenvectors across the graph is measured by eigenvalues. A smaller graph frequency is desired for smooth brain activity.

For an EEG signal at time *t*, the graph Fourier transform is

$$\hat{x(t)}=U^{T}x(t),$$

where

U: matrix of eigenvectors,

$\hat{x(t)}$ = Graph frequency representation of the EEG signal.

**Algorithm 1: GFT(X)**

**Input:**

X: EEG signal (n*t) [n channels * t timepoint]

**Output:** GFT coefficients

**Step 1:** A = correlation(X)

**Step 2:** Calculate degree matrix D of adjacency matrix A

**Step 3:** L = D − A

**Step 4:** Compute eigenvalues $\lambda_{i},i=1:n$

**Step 5: For** i = 1 to n:

Compute eigenvectors *µ_i_*

**Step 6:** Create *U*[*n*][*n*] with eigenvectors as columns

**Step 7:** Compute graph signal representation: $\hat{x\left(t\right)}\;=\;U^{T}x(t)$

### 3.3. Experimental protocol and validation strategy

**Figure 1.**
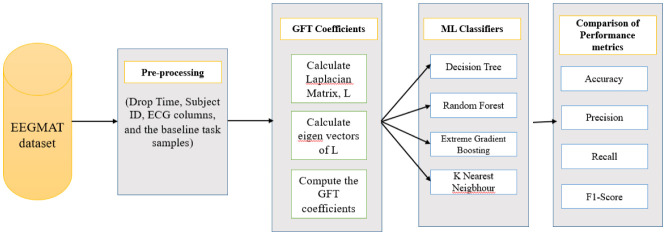
Methodology.

A robust evaluation was ensured employing stratified 5-fold cross-validation strategies, preserving the class distribution. The dataset was divided into 5 nonoverlapping folds on a random basis, where for each iteration, 4 folds were used for training and 1 was used for testing. All preprocessing steps were strictly done on the training folds to prevent any kind of data leakage. The columns of Electrocardiogram (ECG), time, and baseline task were dropped, and a bandpass filtering of 0.5 Hz to 40 Hz was performed. Standardization of the signals is done using z-score normalization, and then the graph based on the Pearson correlation is formed. The correlation threshold is taken to be 0.5, to have a balanced approach. A Pearson correlation was chosen instead of other graph construction strategies, such as phase locking value and mutual information, for computational simplicity and interpretability. The threshold of 0.5 retains only statistically meaningful connections and is consistent with prior works in the field of EEG with graph signal processing. The methodology of this study has been illustrated in Figure 1.

Hyperparameters for all classifiers were fixed to avoid any kind of evaluation bias.

- RF: 200 trees, max-depth = none
- DT: Gini criterion, max-depth = 10
- XGB: learning rate = 0.1, max-depth = 6, n-estimators = 100
- LR: max iterations = 1000, L2 regularization

The performance metrics used for evaluation are accuracy, recall, precision, and F1-score.

## 4. Results

The extracted features using GFT were passed onto different machine learning-based classifiers, RF, DT, XGB, and LR. Table 2 shows the results of all the classifiers and their comparison with the classification results of the same models with raw signals. The accuracy, precision, recall, and F1-score values have been taken as the performance metrics.

**Table 2.** Performance of different classifiers with and without GFT features

	F1-score (raw)	Accuracy (GFT)	Precision (GFT)	Recall (GFT)	F1-score (GFT)
RF	0.98	0.99	0.99	0.99	0.99
XGB	0.80	0.84	0.85	0.84	0.83
DT	0.92	0.94	0.94	0.94	0.92
LR	0.60	0.72	0.52	0.72	0.60

**Figure 2.**
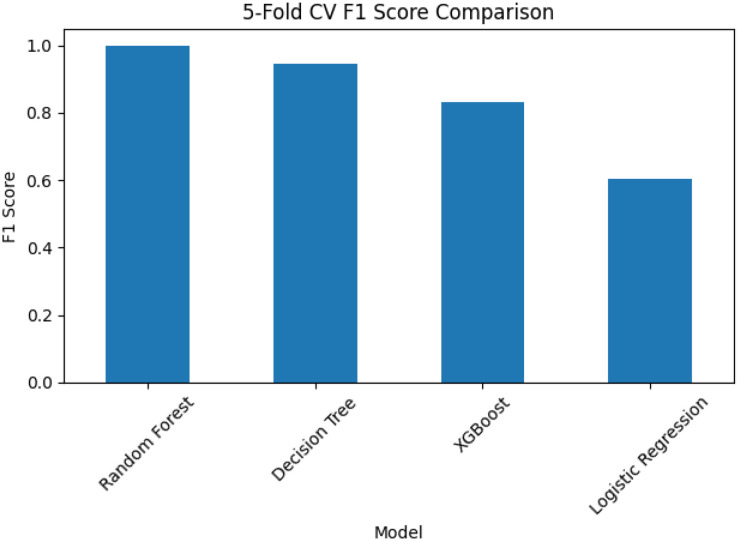
Comparison of F1-scores of the classifiers embedded with GFT-based features.

**Figure 3.**
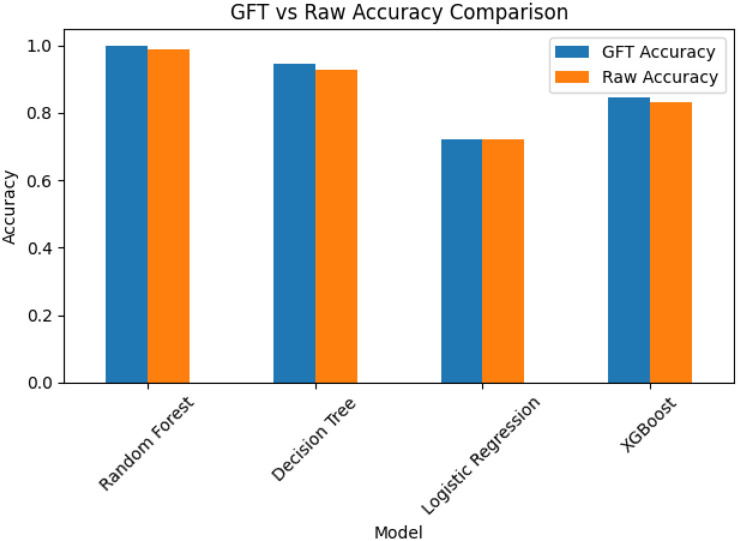
Bar plot representation of accuracies of the classifiers with and without GFT features.

**Figure 4.**
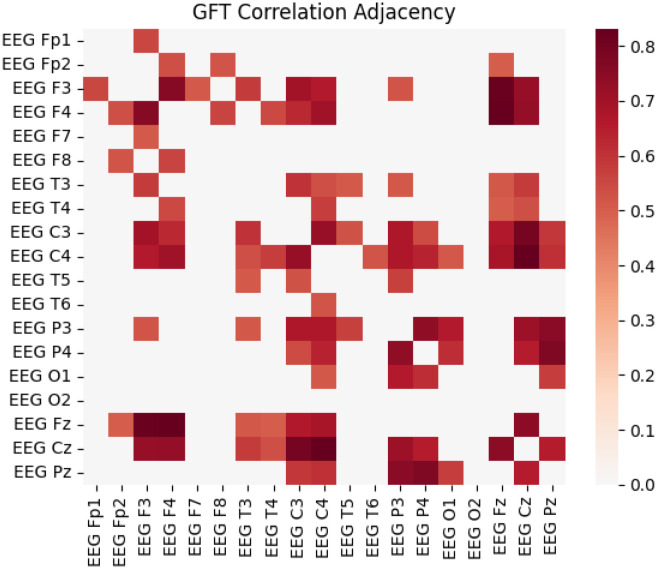
Pearson correlation graph of the channels on the training data.

The F1-scores across the classifiers are represented as bar plots in Figure 2. RF has given the best results while LR has shown the lowest performance. The comparative analysis of accuracies with and without the use of GFT-based features is shown in Figure 3. All the classifiers, except LR, have shown improvement with GFT-based features. As LR is a linear classifier, it has not worked well with the nonlinear EEG dataset or correlation-based graph features. The LR model has been purposefully included in this study to understand the importance of the correct combination of feature extraction technique and the implemented classification model. The pearson correlation graph of the EEGMAT dataset is shown is Figure 4.

### 4.1. Statistical significance analysis

**Figure 5.**
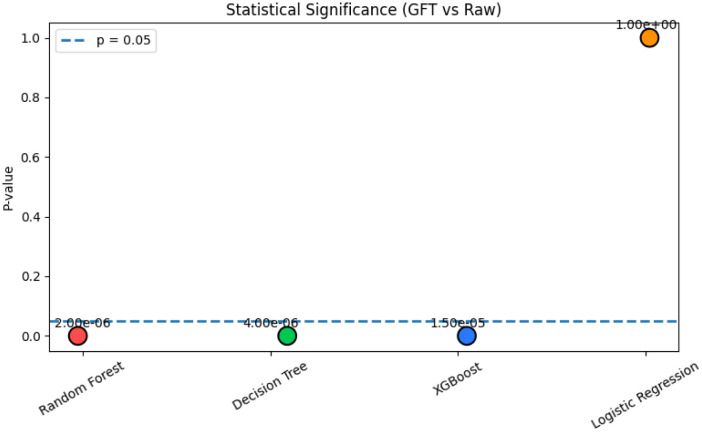
Statistical analysis with p-values.

To validate that the observed improvement in performance of the classifier from GFT features is statistically significant, a Wilcoxon signed-rank test has been conducted across the cross-validation folds for each classifier, comparing each GFT-based result with the result on raw signals. The p-value was assessed at *α* = 0.05. The Figure 5 shows that for tree-based classifiers, the p-value is below 0.05, making the improvements statistically significant. For the linear LR classifier, the performance is low and has not been improved using the correlation-based GFT features.

### 4.2. SHAP based interpretability analysis

**Figure 6.**
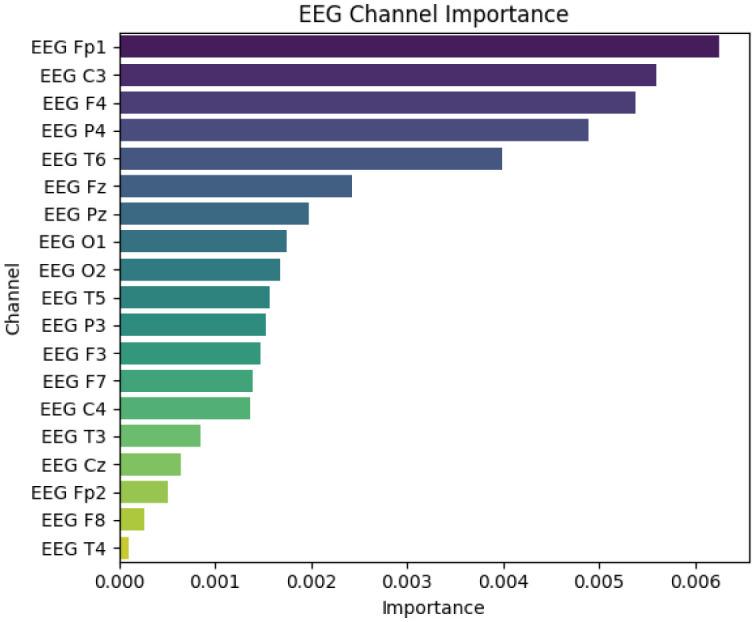
SHAP-based channel importance.

The RF classifier has given the best performance amongst all, and has been chosen for SHAP (Shapely additive explanations) interpretability analysis. SHAP assigns a contribution value to each input feature, identifying the relative importance of individual EEG channels in the classification decision. The SHAP analysis revealed that the frontal (Fp1, F3, Fz) region contributes substantially to classification of cognitive performance in mental arithmetic tasks. These findings align with the well-established fact from cognitive neuroscientists that the frontal region is responsible for working memory, numerical cognition, and executive control functions needed for a serial subtraction task. The C3 channel has high importance pertaining to the central region that coordinates brain activation during attention, and Pz takes care of visual-spatial processing needed while doing mental arithmetic. The channel importance of the dataset in the RF classifier has been shown in Figure 6.

### 4.3. Computational efficiency

The primary motive of this study was to develop a lightweight alternative to computationally heavy deep learning models. The proposed GFT-based feature extraction involves computing a graph Laplacian eigen decomposition of N (N = 19 with *O*(*N*^3^) channels and a matrix vector product per sample. There is negligible inference overhead in comparison to deep learning-based models such as convolutional neural networks (CNN) and recurrent neural networks (RNN). The total number of parameters in the RF model implemented in this study (200 trees) is way fewer than a convolutional or recurrent based network. On a standard intel-i7 processor, the RF pipeline processes each step in under 5 ms. The model is within the time requirements of EEG signal processing for cognitive states.

## 5. Discussion

The experimental results demonstrate an enhancement in the performance of classical machine learning models when combined with the proposed lightweight GFT feature extraction technique. The consistent improvement across all classifiers, especially tree-based learners, indicates that GFT is capable of learning spatial dependencies of the data, which is often overlooked in conventional approaches.

The lower performance of the LR model highlights the inability of the linear model to classify nonlinear EEG data. The higher performance by the tree-based models suggests the suitability of ensemble learning methods to understand the complexity of nonlinear, nonstationary EEG signals. Unlike the traditional signal processing techniques, like short-time Fourier transform (STFT) and continuous wavelet transform (CWT), which work on temporal-frequency characteristics of the data, GFT provides spatial representations of inter-channel relationships for multichannel EEG data. While STFT and CWT have the ability to localize short-duration neural oscillations at a timepoint, they treat each channel independently. GFT works on inter-domains across channels simultaneously and captures inter-regional dependencies of the brain. Hence, the variation across brain regions is effectively captured, providing a meaningful representation of brain activity.

The interpretability analysis using SHAP dives more into the decision-making process of the model. The frontal and central regions are found to contribute the most to classification performance. This observation is consistent with findings of cognitive neuroscience, as the prowess of an individual in a mental arithmetic task will depend highly on his/her frontal brain region. This alignment between classification model and domain knowledge validates the proposed approach on the given dataset.

Despite the good performance, there are limitations of this study which must be addressed. Due to limited data size and few subjects, inter-subject variability could not be tested. Sequential structures, like LSTM and transformers, can also be explored with the GFT features. Further, the graph construction relies on Pearson correlation only whereas other graph construction techniques can be explored in future works. Nonlinear functional connectivity measures, such as mutual information, can generate richer graphs. Future works should also include graph-based neural networks, such as graph neural network (GNN) and graph convolutional neural network (GCNN), to capture spatial-temporal dependencies. Extending the evaluation to larger datasets will help in generalization of the model. Explicit temporal modeling has not been implemented in this dataset, which can be explored in upcoming studies. Future works may also include leave one subject out (LOSO) cross-validation to ensure strict subject-independent evaluation and implementation on other cognitive tasks such as the Stroop test and N-back task.

## 6. Conclusion

This study aims to provide a lightweight and interpretable alternative to computationally heavy deep learning models. GFT-based feature extraction combined with ML models is used for cognitive-state classification using EEG signals. The study demonstrates the improvement of classification accuracy by the use of GFT-based features, with RF achieving the highest accuracy. The statistical analysis confirms the significance of these improvements, particularly in tree-based models. SHAP-based interpretability has given insight into the importance of different brain regions during the given mental arithmetic task.

These findings highlight the importance of graph-based techniques in classification of EEG signals and importance of incorporating spatial dependencies in the analysis of EEG data. The proposed approach gives a balance between performance and computational effectiveness with no requirement of a GPU-based system. There are limitations of data size and number of subjects, as well as the absence of temporal modeling. These limitations will be addressed in the future works. Overall, GFT is established as an effective and lightweight feature extraction technique, which when clubbed with the correct classifier, can give remarkably high classification results.

## Use of AI tools declaration

The authors declare they have not used Artificial Intelligence (AI) tools in the creation of this article.
